# Recent advances in understanding vitiligo

**DOI:** 10.12688/f1000research.8976.1

**Published:** 2016-09-06

**Authors:** Prashiela Manga, Nada Elbuluk, Seth J. Orlow

**Affiliations:** 1 The Ronald O. Perelman Department of Dermatology, New York University School of Medicine, New York, NY, 10016, USA

**Keywords:** Vitiligo, melanocyte, oxidative stress, IFN-γ, Phototherapy

## Abstract

Vitiligo, an acquired depigmentation disorder, manifests as white macules on the skin and can cause significant psychological stress and stigmatization. Recent advances have shed light on key components that drive disease onset and progression as well as therapeutic approaches. Vitiligo can be triggered by stress to the melanin pigment-producing cells of the skin, the melanocytes. The triggers, which range from sunburn to mechanical trauma and chemical exposures, ultimately cause an autoimmune response that targets melanocytes, driving progressive skin depigmentation. The most significant progress in our understanding of disease etiology has been made on three fronts: (1) identifying cellular responses to stress, including antioxidant pathways and the unfolded protein response (UPR), as key players in disease onset, (2) characterizing immune responses that target melanocytes and drive disease progression, and (3) identifying major susceptibility genes. The current model for vitiligo pathogenesis postulates that oxidative stress causes cellular disruptions, including interruption of protein maturation in the endoplasmic reticulum (ER), leading to the activation of the UPR and expression of UPR-regulated chemokines such as interleukin 6 (IL-6) and IL-8. These chemokines recruit immune components to the skin, causing melanocytes to be targeted for destruction. Oxidative stress can further increase melanocyte targeting by promoting antigen presentation. Two key components of the autoimmune response that promote disease progression are the interferon (IFN)-γ/CXCL10 axis and IL-17-mediated responses. Several genome-wide association studies support a role for these pathways, with the antioxidant gene *NRF2*, UPR gene *XBP1*, and numerous immune-related genes including class I and class II major histocompatibility genes associated with a risk for developing vitiligo. Novel approaches to promote repigmentation in vitiligo are being investigated and may yield effective, long-lasting therapies.

## Introduction

Vitiligo affects about 1% of people worldwide and is phenotypically characterized by acquired depigmented patches of skin from which melanocytes (pigment-producing cells) have been lost ^1^. The most common type is non-segmental generalized vitiligo (hereafter referred to as vitiligo), which presents with widely distributed, usually symmetric, and progressive lesions. Vitiligo has a pronounced impact on the physical and mental health of patients, including loss of skin photoprotection, compromised cutaneous immunity, and an appreciable reduction in quality of life that is directly correlated with the early age of onset (typically in the first two decades of life) ^2, 3^.

Recent studies have begun to reveal the pathophysiology of vitiligo. A trigger event is thought to instigate stress responses in the skin that elicit an autoimmune response in genetically susceptible individuals that ultimately targets the melanocytes known to have an inherited fragility, predisposing individuals to develop vitiligo ^4^. The most significant progress in our understanding of disease etiology has been made on three fronts: characterizing the stress responses activated by vitiligo triggers, delineating the autoimmune components that cause disease progression, and identifying susceptibility genes. There are currently no treatments for vitiligo that effectively promote complete repigmentation with long-lasting effects while preventing recurrence. Total depigmentation therapy using monobenzone (for severe cases) is currently the only treatment approved by the US Food and Drug Administration (FDA) for vitiligo; novel approaches to prevent further loss and promote repigmentation are being investigated and may yield effective, long-lasting therapies. These efforts are supported by global collaborations that have drawn up consensus guidelines for disease classification ^5^, categorizing severity ^6^, and outcome measures that are also useful for clinical studies ^7^.

## Stress responses in vitiligo

The onset of vitiligo can be instigated by various triggers, including sunburn and exposure to phenolic chemicals; however, the trigger is not known in most cases. The triggers are all thought to induce oxidative stress in melanocytes ^8^. Individuals with vitiligo have been reported to have compromised antioxidant responses ^9^, with enzymes such as superoxide dismutase (SOD) present at higher-than-expected levels in tissue from perilesional areas and in sera ^10^. The key role of antioxidants in vitiligo has been suggested by a candidate gene association study, which found a significant association between single nucleotide polymorphism (SNP) rs3565214 within *NRF2* and vitiligo in the Chinese population ^11^.

There are multiple mechanisms through which excessive melanocyte oxidative stress can translate to an autoimmune reaction. For example, the antioxidant SOD has been linked to vitiligo, with increased expression in tissues from patients, and genetic linkage of isoforms 2 and 3 with increased susceptibility ^12^. The antioxidant response also promotes the expression of inducible heat shock protein 70 (iHSP70), which can serve multiple roles in the cellular stress response, including targeting the antioxidant SOD-2 to mitochondria ^13^.

Oxidative stress can also lead to increased iHSP70 secretion, which has been documented in vitiligo melanocytes ^14^ and may provide a novel therapeutic target, since overexpression of iHSP70 in the skin has been shown to cause melanocyte loss in mice ^15^. Gene gun vaccination of mutant HSP70 before depigmentation in a vitiligo mouse model (mice expressing Pmel-1/gp100-reactive T cell receptor, resulting in melanocyte loss) prevented vitiligo, while depigmentation was reversed in a second model (mice expressing a tyrosinase-reactive T cell receptor, resulting in melanocyte loss) undergoing rapid pigment loss ^14^.

Melanocytes cultured from vitiligo patient skin samples were found to demonstrate changes in signal-transduction pathways such as mitogen-activated protein kinase hyperactivation and increased sensitivity to apoptosis inducers, which may be the result of sustained but sublethal oxidative stress. These melanocytes also expressed high levels of cytokines including interleukin-6 (IL-6), matrix metalloproteinase-3, and insulin-like growth factor-binding protein-3 and -7 ^16^. Picardo *et al*. ^17^ suggest that this constitutes a “senescence phenotype” characterized by the secretion of cytokines that provoke an autoimmune response similar to that seen in neurodegenerative disease.

Oxidative stress extends to the endoplasmic reticulum (ER), which is frequently dilated in perilesional melanocytes from patients with vitiligo ^18^. The ER is a sensor of cellular stress and the site of protein maturation, which requires a regulated environment to facilitate the chemical bond formation required for secondary and tertiary protein structure. Disruption of the ER redox balance results in the accumulation of misfolded proteins, which in turn activates the unfolded protein response (UPR) ^19^. The UPR ameliorates ER stress by signaling a transient halt in global protein synthesis, increasing the expression of chaperones that facilitate protein folding, and increasing the degradation of misfolded proteins; however, sustained activation results in apoptosis ^20^. Melanocytes can, however, adapt to continual UPR activity and evade UPR-induced death ^21, 22^. Chemical agents that trigger vitiligo, such as phenolics (4-tertiary-butylphenol and monobenzylether of hydroquinone), induce oxidative stress and promote UPR activation ^23^. UPR-induced expression of cytokines, such as IL-6 and IL-8, can attract immune components to the skin. The UPR may thus be the link between a trigger event and the initiation of an autoimmune response that results in vitiligo progression. Interestingly, the UPR also contributes to the activation of the immune response ^24^ and plays a role in autoimmune disorders ^25^ such as type I diabetes ^26^ and neurodegenerative disorders ^27^. Genome-wide linkage analysis followed by a sequencing study in a Chinese population with vitiligo identified X-box binding protein 1 ( *XBP1*) ^28, 29^, which was then confirmed in a vitiligo Caucasian cohort ^30^; this gene encodes a transcription factor that mediates UPR activation ^31^.

## The autoimmune component

Autoimmunity has long been suspected to feature significantly in the pathogenesis of vitiligo, and multiple studies published in the past few years increasingly shed light on the role of the immune system in vitiligo. CD8 ^+^ T cells specific to, and capable of killing, melanocytes are increased in the blood of those with vitiligo compared to healthy controls, and numbers correlate with disease activity. Using an engineered mouse model of vitiligo, Harris and co-workers had previously found that interferon (IFN)-γ played a central role in the spread of vitiligo lesions ^32^. Specifically, they showed that IFN-γ caused an increase in the expression of CXCL10, a chemokine which regulates the invasion of epidermal and follicular tissues by CD8 ^+^ T cells. IFN-γ was also identified as part of a “signature cytokine profile” in an avian model of vitiligo. The Smyth line (SL) of chickens develops a spontaneous, depigmentation disorder that shares several key clinical and pathologic features with human vitiligo. For example, melanocytes that pigment the feathers are lost in an autoimmune-driven process. As the disease progresses, there is an increase in the expression of IFN-γ ^33^. Recently, however, a study by Yang *et al*. suggested that IFN-γ could play an even more direct role in vitiligo pathogenesis by demonstrating that the IFN-γ derived from cytotoxic T cells could itself cause apoptosis in melanocytes ^34^. An accompanying editorial by Harris clearly puts Yang’s group’s results into the larger context ^35^.

IL-17 and T helper type 17 (Th17) cells, which elaborate this cytokine, have been increasingly recognized to play an important role in autoimmunity. The potential role of Th17 in vitiligo has recently been reviewed comprehensively by Singh and colleagues ^36^. They discuss recent studies in which blood, tissue, and cellular levels of IL-17 have been found to be elevated in vitiligo. Positive correlations between levels of IL-17 and disease extent and activity have also been found. A recent example that illustrates this is the work of Zhou *et al*. ^37^. They found that levels of Th17 cells (as well as the cytokines transforming growth factor [TGF]-β and IL-21, which matched the findings in the SL avian model, where the expression of IL-21 ^33^ and its receptor, IL-21R, increased as the disease progressed ^38^) correlated with disease activity in generalized vitiligo. Singh *et al*. also discussed how treatments that improve vitiligo, such as ultraviolet B (UVB) phototherapy, may also modulate IL-17 levels. These findings are particularly exciting in view of the increasing availability of biologic therapeutics that target the IL-17 axis.

While the ability to inhibit IL-17 in the clinical sphere is relatively new, inhibitors of TNF-α have been available for over a decade. Some (but not all) studies previously published have shown an increase in tumor necrosis factor (TNF)-α associated with vitiligo. Webb *et al*. ^39^ observed that TNF-α inhibition was associated with the blockade of progression in three vitiligo patients and pointed out that this effect might have been missed previously because past studies focused on the ability of TNF-α blockade to promote repigmentation, a very different endpoint. They also noted the paradoxical onset of vitiligo reported in some patients with other autoimmune diseases treated with TNF-α inhibitors. This phenomenon has also been observed in psoriasis, where TNF-α inhibitors ushered in the modern era of efficacious psoriasis therapy, yet sometimes psoriasis can develop *de novo* in patients with another autoimmune disease like rheumatoid arthritis or inflammatory bowel disease treated with a TNF-α inhibitor.

The increased levels of IL-21 noted above by Zhou *et al*. ^37^ raise the question of the involvement of a newly recognized subset of T cells, called follicular helper T (Tfh) cells, in vitiligo. Tfh cells can be distinguished from Th17 cells by both their production of IL-21 and their ability to home to B cell areas in secondary lymphoid tissue. Tfh cells and IL-21 are believed to play a central role in B cell activation but are increasingly recognized as possible players in the immune dysregulation that typifies autoimmunity. Furthermore, IL-21 has been shown to be critical in the pathogenesis of murine autoimmune diabetes ^40^ and promotes an increase in CD8+ T cells ^41^ and mediates prolonging of their cytotoxic responses ^42^.

The class I and class II major histocompatibility loci located on chromosome 6p21.3 have been associated with a variety of autoimmune diseases, including vitiligo. Some such associations could point to a preferential presentation by cells of certain antigens—for example, melanocyte-specific antigens like tyrosinase—to the immune system. Recently, through a genome-wide association study of 2,853 Caucasian vitiligo patients ^43^, Spritz, Dinarello, and colleagues found three SNPs located within a predicted super-enhancer in an intergenic region between the HLA-DRB1 and HLA-DQA1 loci. The super-enhancer correlated with increased expression of both major histocompatibility complex (MHC) class II proteins on monocytes from normal volunteers homozygous for the high-risk haplotype. They found that upon stimulation of monocytes with microbe-derived products, the production of both IFN-γ and IL-1β was 2.5- and 5-fold higher, respectively, in those with the high-risk haplotype than it was in those homozygous for the low-risk haplotype, thereby providing a potential link between the level of MHC class II expression and the elaboration of cytokines that could provoke or perpetuate an autoimmune response. Spritz and colleagues also identified a haplotype at the MHC class I locus that is associated with vitiligo susceptibility. The haplotype spans a region that includes a transcriptional regulator downstream of the *HLA-A* gene, and carriers of the vitiligo-associated haplotype were found to express higher levels of HLA-A RNA transcript compared to carriers of the non-vitiligo-associated haplotypes ^44^. Vitiligo susceptibility is also associated with the HLA-A *02:01:01:01 allele ^45^, which encodes HLA-A2 and can present melanocyte protein-derived autoantigens. When the vitiligo-associated risk haplotype/alleles are present in combination, the regulatory region risk haplotype drives elevated expression of HLA-A2 and thus increased presentation of melanocyte-specific proteins that are readily recognized by cytotoxic T cells ^44^.

## Advances in vitiligo treatments

Therapeutic options available for stabilizing and repigmenting vitiligo have been modestly expanded in recent years, although only depigmentation therapy using monobenzone is approved by the FDA. Depigmentation therapy is reserved for the treatment of remaining normal skin in those with extensive vitiligo affecting the majority of one’s body. Traditional therapies for repigmentation, including topical agents and phototherapy, remain mainstays of current treatment. Topical treatments include corticosteroids, calcineurin inhibitors, and vitamin D analogues ^46– 49^. Phototherapy treatments include narrowband UVB (NB-UVB) or psoralen and UVA (PUVA) ^46– 49^. NB-UVB, which consists of 311–313 nm, can be given to the whole body using lamps or as a focused, targeted treatment using a 308 nm xenon-chloride monochromatic excimer light emitted through a laser or incoherent lamp ^50– 52^. More recently, studies have evaluated other types of phototherapy such as broadband UVB (280–320 nm), psoralen combined with NB-UVB, UVA-1, and PUVA sol ^53– 56^. Newer studies of traditional treatments have compared the use of these treatments as monotherapies as well as evaluated the efficacy of combining treatments for a multimodal approach ^57– 60^. Several studies have found that phototherapy combined with topical creams yield faster and greater repigmentation than each treatment modality as a monotherapy ^57, 59, 60^. While the combination approach has led to successful repigmentation for many patients, there remain many individuals who have unsatisfactory results and for whom alternative treatment options are needed. Advances in vitiligo therapy have sought to investigate these alternative treatments, which include topical, oral, and procedural treatments that seek to target different pathways involved in the pathogenesis of vitiligo.

### Topical treatments

Prostaglandin analogues traditionally used for glaucoma therapy have been found to induce hyperpigmentation through effects that lead to melanocyte proliferation ^61^. Several studies have evaluated the use of topical prostaglandin analogues for the treatment of localized vitiligo ^61– 63^. Two different studies evaluating the use of topical prostaglandin E2 on localized areas of vitiligo for 6 months resulted in moderate to complete repigmentation in the majority of patients ^62, 63^. Another study found that topical latanoprost was found to result in comparable results to NB-UVB and, when the two therapies were combined together, it led to greater repigmentation ^64^. Larger, randomized controlled trials and comparative studies with other treatment alternatives are needed to better determine the efficacy and safety of prostaglandin analogues for vitiligo repigmentation.

### Oral and systemic treatments

Oral corticosteroids have often been used for short durations in rapidly spreading vitiligo; however, caution must be taken given the potential side effects of systemic steroids. Studies have sought to find lower strengths of steroids, which can still provide benefit in halting the progression of vitiligo. Kanwar *et al*. found that low-dose oral dexamethasone mini pulse therapy of 2.5 mg per day on two consecutive days of the week halted vitiligo in 91.8% of subjects in roughly 13 weeks ^65^.

Minocycline, a broad-spectrum antibiotic, has also been evaluated in vitiligo owing to its anti-inflammatory, antioxidant, and immunomodulatory properties ^66– 68^. Minocycline’s ability to scavenge free radicals has been found to have a protective effect on melanocytes against H _2_O _2_-induced apoptosis ^69^. A clinical study in which vitiligo patients were given minocycline 100 mg once daily showed arrest of disease progression in 91% of patients ^67^. A randomized clinical trial compared treatment with 6 months of minocycline to oral mini pulse corticosteroids and found that the two treatments were comparable in stopping the progression of actively spreading vitiligo with minimal side effects in each group ^68^.

Oral statins, traditionally used to lower cholesterol, have also been evaluated in vitiligo treatment because of their immunomodulating and antioxidant properties. In addition to scavenging free radicals, statins lead to the downregulation of inflammatory cytokines including IL-6, IL-2, IFN-γ, and TNF-α ^70, 71^. Regression of vitiligo was first reported in a case report of a man with vitiligo who was taking high-dose oral simvastatin for treatment of high cholesterol ^72^. Agarwal *et al*. found that in a mouse model of vitiligo, statins could prevent and reverse depigmentation of vitiligo by decreasing IFN-γ production and by stopping the influx and proliferation of cutaneous autoreactive CD8+ T cells ^73^.

Agents which are commonly used to treat rheumatologic diseases and psoriasis are also being studied in the treatment of autoimmune diseases including vitiligo. Janus kinase (JAK) inhibitors have been evaluated in several case reports, with potential therapeutic promise thought to be related to their interference with IFN-γ signaling ^48, 49, 74, 75^. Research has shown that IFN-γ-induced expression of CXCL10 is critical for the progression and maintenance of depigmentation in vitiligo ^76^. In a recent case report, a patient with vitiligo was treated with tofacitinib, an oral JAK 1/3 inhibitor, for 5 months, and had significant repigmentation with no significant adverse effects ^74^. Ruxolitinib, another JAK inhibitor, was studied in a patient with alopecia areata and vitiligo and was found to lead to 51% facial repigmentation compared to 0.8% at baseline ^75^. However, 12 weeks after discontinuation of the drug, the patient had lost much of the pigment he had regained.

Oral vitamins and supplements have also gained increased interest in the treatment of vitiligo owing to their antioxidant properties. L-phenylalanine, khellin, polypodium leucotomos, *Ginkgo biloba*, B12, folic acid, vitamins C and E, alpha lipoic acid, and zinc have all been studied either as monotherapies or in combination with other treatments with varying efficacy in improving vitiligo repigmentation ^3, 77– 84^. Though several of these treatments are promising, the majority of these studies have been done in a small number of patients and many without controls. Larger randomized controlled trials are needed to make more definitive treatment recommendations and to better understand how these agents can reverse depigmentation.

Afamelanotide, a longer-acting synthetic analogue of alpha-melanocyte-stimulating hormone, has also shown promise in early clinical studies ^59, 60, 85, 86^. By binding to the melanocortin-1 receptor, afamelanotide may combat melanocortin system defects in vitiligo patients by stimulating melanocyte proliferation and melanogenesis. An early phase clinical trial found that when patients were given an implant of 16 mg of afamelanotide and then had additional NB-UVB treatment, the combination yielded faster and more extensive repigmentation of facial and upper extremity vitiligo lesions than did patients who received only NB-UVB ^86, 87^. Adverse effects of this treatment include nausea, fatigue, abdominal pain, and hyperpigmentation.

### Procedural treatments

Several procedural treatments using laser and surgical techniques may also provide hope for patients with stable vitiligo who have not repigmented with traditional therapies. Erbium laser-assisted dermabrasion and fractional CO _2_ lasers have been used on vitiligo patients followed by NB-UVB and have been found to result in superior repigmentation compared with the use of NB-UVB alone ^88, 89^. However, the pain, scarring, and healing time that can be associated with ablative laser therapy may preclude this from becoming a more mainstream treatment option.

Additional surgical options for vitiligo treatment include autologous punch and suction blister grafts, split thickness grafts, needling, and non-cultured epidermal cell suspension also known as melanocyte keratinocyte transplantation (MKTP) ^90– 95^. The latter technique involves the application of an autologous cell mixture to an abraded recipient site. This is often followed by continued treatment with phototherapy. Improvements to this technique have made it an effective and well-tolerated procedure with high repigmentation rates ^96, 97^.

## Conclusion

Current medical and surgical therapies for vitiligo, particularly when used in combination, have shown some success in the stabilization and repigmentation of vitiligo. New therapies are on the horizon, and the future for vitiligo is promising. In addition, international collaborations to establish common outcome criteria will support these efforts ^7^. Continued research into the pathogenesis of this complex and multifactorial disease will help provide further insight into disease targets and how to best approach treatment.

## Abbreviations

ER, endoplasmic reticulum; FDA, US Food and Drug Administration; IFN, interferon; iHSP70, inducible heat shock protein 70; IL, interleukin; JAK, Janus Kinase; MHC, major histocompatibility complex; NB-UVB, narrowband UVB; NRF2, nuclear factor erythroid 2-related factor 2; PUVA, psoralen and ultraviolet A; SL, Smyth line; SNP, single nucleotide polymorphism; SOD, superoxide dismutase; Tfh, follicular T helper; TH17, T helper type 17; TNF, tumor necrosis factor; UPR, unfolded protein response; UVB, ultraviolet B.
